# Hawthorn Juice Simulation System for Pectin and Polyphenol Adsorption Behavior: Kinetic Modeling Properties and Identification of the Interaction Mechanism

**DOI:** 10.3390/foods11182813

**Published:** 2022-09-13

**Authors:** Xuan Zhang, Meijiao Li, Wen Zhao, Zhe Gao, Mengying Wu, Tong Zhou, Chen Wu, Kaixuan Zhou, Xue Han, Qian Zhou

**Affiliations:** 1 College of Food Science and Technology, Hebei Agricultural University, Baoding 071000, China; 2 Engineering Technology Research Center for Agricultural Product Processing of Hebei, Baoding 071000, China

**Keywords:** hawthorn, pectin, epicatechin, chlorogenic acid, non-covalent interaction

## Abstract

The interaction between polyphenols and polysaccharides plays an important role in increasing the turbidity stability of fruit juice and improving unpleasant sensory experiences. The binding adsorption behavior between hawthorn pectin (HP) and polyphenols (epicatechin and chlorogenic acid) accorded with the monolayer adsorption behavior driven by chemical action and were better fitted by pseudo-second order dynamic equation and Langmuir model. The HP binding sites (Q_m_) and adsorption capacity (Q_e_) to epicatechin were estimated at 75.188 and 293.627 μg/mg HP, respectively, which was about nine and twelve times higher than that of chlorogenic acid. The interaction between HP and polyphenols exhibited higher turbidity characteristics, particle size and lower zeta potential than epicatechin and chlorogenic acid alone. Meanwhile, according to Fourier Transform Infrared Spectroscopy (FT-IR) analysis, it could be speculated that the interaction between HP and polyphenols resulted in chemical combination. Moreover, ΔH < 0 and TΔS < 0, which indicated that the interaction between HP and polyphenols was mainly driven by hydrogen bonds and van der Waals forces.

## 1. Introduction

Hawthorn, belonging to the *Crataegus* spp. of *Rosaceae* family, is a deciduous and fruit-bearing shrub or small tree which is originated and distributed in East Asia, Europe and North America with 260 species [1,2]. As a kind of edible fruit, hawthorn has a long history which might be dated back to more than three thousand years ago recorded in the ancient books *Erya* in China. Nowadays, besides the fresh-eating, hawthorn would be processed as snacks, drinks and fillings as well as available as dietary supplements in the food market. As a kind of hawthorn processing product, hawthorn juice is favored by consumers due to its high nutritional value which may be associated with its bioactive components, such as high pectin content, polyphenols and other bioactive substances [1,3]. However, the formation of turbidity and unpleasant sensory experiences such as bitterness and astringency were considered a common phenomena in the juice production process and pectin could well improve unpleasant sensory experiences by the interaction between polyphenol [4,5,6]. It should be noted that in different systems, the interaction between pectin and polyphenol was different, and the influence on food systems such as fruit juice was also different. Therefore, exploring the relationship between pectin and polyphenol could provide a theoretical basis for improving the quality of fruit juice.

Polyphenols are generally localized in vacuoles of the living plant cells and they would be released in the food processing in which there are various external forces such as heating, drying and high pressure [7,8]. Additionally, multiple food matrix ingredients could also be released and contacted with polyphenols quickly and spontaneously, which would affect the bioavailability of polyphenols [9,10]. Diez-Sanchez et al. have shown that when polyphenols were incorporated into the model systems (dietary fiber, protein, etc.), the bioaccessibility was increased compared to polyphenols alone [11]. Liu et al. also have shown that dietary fiber and other food matrix could be used as a carrier for polyphenols, which could not only improve the bioavailability of polyphenols, but also transport these antioxidants to the human gut microbiota and further produce beneficial physiological effects after their fermentation in small phenolic compounds [12]. Pectin is an anionic polysaccharide from cell wall and mainly composed of linear chains of galacturonic acid. Depending on the composition, the structure of pectin is categorized into three main structural units, namely homogalacturonan (HG), rhamnogalacturonan-I (RG-I) and rhamnogalacturonan-Ⅱ (RG-Ⅱ) [13]. Furthermore, as an essential constituent in food, pectin could form complex with polyphenols and the complex systems have received an increasing research attention in order to extend their food applications in recent years.

Pectin had the stronger interaction with polyphenols, which might be dependent on their structural characteristics (e.g., molar mass, size, proportion of branching length and degrees of methylation) and was dominated by noncovalent interactions driven by hydrophobic and hydrogen bonds [12,14]. Strong binding was observed between highly galloylated tannin and pectin with high degree of esterification from persimmon, which was controlled by cooperative hydrogen bonding and hydrophobic interaction [15]. Hairy regions of pectin with only monomeric side chains showed higher association with procyanidin and involved hydrophobic interaction and hydrogen bond [16]. Higher anthocyanins binding ability was attributed to the more linear, more negatively charged homogalacturonan region of chelator-soluble pectin compared to water-soluble pectin, whereas hydrogen bond and hydrophobic interaction were the main driving forces involved [17]. Furthermore, pectin with different structural compositions showed various binding capacity to polyphenols and this interaction was selective. The binding ability of pectin and proanthocyanidins was in the following order from low to high: arabinans, arabinans + galactans II, galactan I, arabinans + galactan I + xylogalacturonans and rhamnogalacturonan [16]. Isothermal titration calorimetry indicated association constants (Ka) of interactions between pectin from citrus peel and persimmon tannin presented range from 2.04 × 10^3^ to 8.50 × 10^3^ M**^−^**^1^ [15], whereas the strong affinity (Ka = 1.4 × 10^4^ M**^−^**^1^) was between apple pectin and procyanidin [14]. Similar findings were also confirmed in studies of pectin and proanthocyanidins derived from grape and pear [14,18].

Interactions between pectin and polyphenols would affect the quality of products [19,20]. Studies have shown that pectins from different sources have different structures, which would determine the interaction with polyphenols and have different effects on the juice system. The grape pectins could affect the aggregation of tannin and protein, which might improve the astringency during fruit processing and beverages production [6]. Symoneaux et al. have shown that pectin could reduce the astringency of fruit wine produced by the interaction between tannin and protein in the mouth [21]. Apple pectin could compete with protein for the binding of proanthocyanidin monomer catechin in the simulated apple juice system, which prevented the system from forming larger particles and enhanced the stability of the system [22]. Pectin of citrus, apple and beet enhance the stability of anthocyanin in blackcurrant and strawberry, which improved color stability of anthocyanins-containing foods and beverages [23,24]. In precision medicine and other field, pectin was also extensively used to encapsulate procyanidins and controlled release according to its hydrogel and porous characteristics [15,23]. However, there was little information about the interaction mechanism between pectin and polyphenol in hawthorn juice system. Therefore, a deep understanding of the driving molecular mechanisms of the interaction between pectin and polyphenol could lead to a better utilization of the benefits of the active ingredients, which was more conducive to the processing and production of fruit juice.

Epicatechin and chlorogenic acid are flavan-3-ol and phenolic acid, respectively. Studies have shown that there were a higher content for epicatechin and chlorogenic acid in Chinese hawthorn [25,26]. Furthermore, based on our previous studies, the contents of epicatechin and chlorogenic acid were preliminarily determined as 269.3 and 78.6 mg/100 g DW in hawthorn fruit (Big Venus), respectively. Based on above, in the current study, epicatechin and chlorogenic acid were applied in the hawthorn juice simulation system as the representative polyphenols for the study of the interaction between polysaccharides and polyphenols. The objectives of our study are (1) to investigate the adsorption capacity between HP and two polyphenols, and (2) to analyze and study the internal driving mechanism of interaction.

## 2. Materials and Methods

### 2.1. Materials

Hawthorn of the variety “Big Venus” was purchased in local supermarket (Baoding, China) at a fully mature ready-to-eat stage. Polyphenols including epicatechin (≥96%) and chlorogenic acid (98%), monosaccharides standards (D-galacturonic acid (98%), D-glucose (98%), D-mannose (98%), D-rhamnose (98%), D-galactose (98%), D-fucose (98%), D-arabinose (98%)), cellulase (400 U/mg), D3520 macroporous resin, phenol and 3-phenylphenol were obtained from Shanghai Yuanye Bio-Technology Co., Ltd. (Shanghai, China). All other chemicals and reagents were of analytical grade and obtained from Baoding Qianyue Trading Co., Ltd. (Baoding, China).

### 2.2. Hawthorn Pectin (HP) Extraction and Purification

The extraction of HP was based on the method described by Guo et al. [27] with a few modifications. After cleaning and removing seeds, fresh hawthorn was cut into 1–2 mm thickness and freeze-dried (4 °C, 20 h). Freeze-dried hawthorn was ground into powder using an electric grinder and sieved with 100-meshes to obtain hawthorn flour. 20 g of hawthorn flours and cellulase (0.6 g) were dissolved in 200 mL distilled water and mixed well. The mixture was then placed into a water bath at 55 °C and extracted for 5 h under constantly and gently stirring. After extraction twice, a process of centrifugation (2273× *g*, 3 min) was followed and the supernatant was combined and cooled to room temperature as the crude HP.

Next, the crude HP was deproteinized with 1/4 volume of Savage reagent (butyl alcohol:chloroform = 1:4) and decolorized using the pretreated D3520 macroporous resin in a 1:10 (g:mL) ratio. Subsequently, the filtrate was rotary evaporated to 1/6 of the initial volume and then three-fold volume of absolute ethanol was added for precipitating the pectin overnight at 4 °C. The precipitate was obtained by centrifugation (2273× *g*, 3 min) to remove ethanol, and then re-dissolved in distilled water and evaporated at 55 °C in order to remove the extra ethanol. The purified HP was definitively obtained by freeze-drying (4 °C, 20 h) and stored in the desiccator for the further analysis.

### 2.3. Chemical Composition Analysis

#### 2.3.1. Monosaccharide Determination

The monosaccharide composition of HP was determined by the method with a few modifications [28]. Briefly, 1 mg/mL of HP aqueous solution mixed with 1 mL of trifluoroacetic acid (TFA, 4 M) was hydrolyzed at 110 °C for 2 h in a 5 mL ampoule bottle. Subsequently, 500 μL of methanol was added to the polysaccharide hydrolysates and it was dried with a nitrogen blower. The residue was dissolved in 1 mL of deionized water and was mixed with 120 μL of NaOH solution (0.3 M) and 120 μL of 1-phenyl-3-methyl-2-pyrazolin-5-one (PMP) methanol solution. The mixture was derivatized in 70 °C water bath for 2 h. Afterwards, the reaction system was neutralized with 120 μL of HCl solution (0.3 M) and extracted three times with 2 mL of chloroform. Then the aqueous phase was filtered through a 0.45 μm filter and analyzed by high performance liquid chromatography (HPLC) equipped with a UV-Vis detector (Agilent 1260). The analysis was performed at 30 °C with a Hypersil BDS (Thermo)-C18 (250 mm × 4.6 mm, 5 μm) at 250 nm. The HPLC solvents composed of 0.1 M phosphate buffer (eluent A) and acetonitrile (eluent B) with the volume ratio was 82:18 (*v/v*), at a 0.8 mL/min flow rate and the injection volume was 20 μL.

#### 2.3.2. Uronic Acid Determination

Uronic acid content was analyzed according to the colorimetric method reported by Kim et al. [29] and applied with a few modifications. 1 mg/mL of HP was mixed with 5 mL of sodium tetraborate in sulphuric acid (12.5 mM) and incubated in a boiling water bath at 100 °C for 5 min. After incubation, the sample was cooled to room temperature and 200 μL of 1.5 mg/mL 3-phenylphenol was added to react with the released uronic acids. Afterwards, the mixture was mixed well before the observation by UV-VIS spectrometer (UV-1800, Shimadzu, Japan) at 525 nm. Control sample was mixed with equal volume of distilled water and incubated under the same conditions. The total content of uronic acid was calculated based on calibration with galacturonic acid standards (20–70 μg/mL). All measurements were done in triplicate.

#### 2.3.3. Total Sugar Determination

Total sugar content was determined by a phenol sulfuric acid method [6] and applied with a few modifications. HP was dissolved in distilled water (1 mg/mL) and diluted 40 times. Afterwards, 5% phenol in sulfuric acid was sequentially added into 1 mL of the diluted solution. After 30 min incubation at room temperature, absorbance was measured at 490 nm in an UV-VIS spectrometer (UV-1800, Shimadzu, Japan). Control sample was mixed with equal volume of distilled water and incubated under the same conditions. The total sugar were measured based on calibration with glucose standards (20–100 μg/mL). All measurements were done in triplicate.

#### 2.3.4. Degree of Methyl Esterification Determination

The degree of methyl esterification (DE) of HP was determined by following the method of Yu et al. [30] with a few modifications. HP (0.2 g) was placed in an erlenmeyer flask and wetted with 1 mL of ethanol, followed by 20 mL of distilled water was added and incubated at 35 °C as well as constantly stirring until dissolved completely. The resulting solution was titrated with 0.1 M NaOH in the presence of phenolphthalein until the solution color changed and the volume of the consumed solution was recorded as V_1_. Then, 10 mL of 0.1 M NaOH solution was added and stirred at room temperature for 2 h. Afterwards, 10 mL of 0.1 M HCl was added and titrated with 0.1 M NaOH until the solution color changed. The volume of the consumed solution was recorded as V_2_. The degree of methyl esterification (DE) of HP was calculated according to the following formula:

DE (%) = (V_2_/(V_1_ + V_2_) × 100 (1)

#### 2.3.5. High Performance Gel Permeation Chromatography

The molecular weight of HP was determined by high performance gel permeation chromatography (GPC) coupled with a refractive index detector (RID-20, Shimadzu, Kyoto, Japan). A guard column of TSK (35 mm × 4.6 mm i.d.), and columns of TSKgel G3000PWXL (300 mm × 7.8 mm i.d.) and TSKgel G4000PWXL (300 mm × 7.8 mm i.d.) (Tosoh Co., Ltd., Tokyo, Japan) were used. HP was dissolved by distilled water (20 mg/mL) and filtered through a 0.45 μm filter. The injection volume was 20 μL, and mobile phase was 0.05 M sodium nitrate solution containing 0.5 g/L of sodium azide as preservative. Elution was carried out at a flow rate of 0.6 mL/min at 35 °C. 1 mg/mL of pullulan polysaccharide (shodex Company, Yokohama, Japan) standard was used as markers [31].

### 2.4. Adsorption Experiments

Adsorption experiments were conducted following the method described by Koh et al. [17] with a few modifications. 0.1 M citrate/phosphate buffer (pH 3.8) hawthorn juice simulation system was established to investigate the interaction mechanism between HP and main polyphenols (epicatechin and chlorogenic acid) in hawthorn. For studying the adsorption kinetics of different incubation times, 0.4 mg/mL of HP and 0.08 mg/mL of epicatechin (EC) and chlorogenic acid (CA) were prepared in 0.1 M citrate/phosphate buffer (pH 3.8), respectively. Then, equal volumes of HP and polyphenol solutions were mixed in dark plastic tubes for different time intervals from 1 min to 24 h. Tubes were constantly shaken at 100 r/min on an orbital shaker at room temperature.

For studying the adsorption isotherm, equal volumes of 0.4 mg/mL of HP were mixed with EC and CA solutions with different concentrations from 0.04–0.2 mg/mL and incubated at room temperature for 4 h. By changing the conditions of pH (2–6), temperature (20–40 °C) and ionic strength (0.025–0.2 M), the influence of adsorption capacity was explored. Unless stated otherwise, all experiments were carried out at room temperature, using 0.1 M citrate/phosphate buffer (pH 3.8) with an incubation time of 4 h. Control samples were mixed with equal volumes of 0.1 M citrate/phosphate buffer (pH 3.8) without HP and incubated under the same conditions.

After incubation, the samples were filtered through a 0.2 μm filter and then detected at the corresponding maximum absorbance wavelengths. The amount of free polyphenols (FP) was defined based on their external standard curves for individual phenolic compounds and bound phenolic compounds (BPC) were calculated using the following equation:

BPC = ((C_0_ − C_FP_) × V)/m (2)

where BPC is bound phenolic compound (μg/mg HP), C_0_ is initial polyphenol concentration (μg/mg), CFP is free polyphenol concentration (μg/mg), V is filtrate volume (mL), m is the mass of HP (mg). All adsorption experiments were performed in triplicate.

### 2.5. Adsorption Model Fitting

#### 2.5.1. Adsorption Kinetics Model Fitting

Intragranular diffusion equation. The model is used to roughly understand the adsorption mechanism and can be described as followed:

Q_t_ = K_p_ t^0.5^ (3)

where Q_t_ is the amount of polyphenols adsorbed by HP at a certain time (μg/mg HP), K_p_ is the rate constant of intragranular diffusion (μg/(mg·min^0.5^)), t is adsorption time (min).

Pseudo-first order dynamic equation. The equation is an adsorption model based on physical adsorption and can be described as followed:

ln (Q_e_ − Q_t_) = ln Q_e_ − K_1_ t (4)

Pseudo-second order dynamic equation. The adsorption equation is mainly characterized as the adsorption process controlled by the chemical action. The equation is:

t/Q_t_ = 1/(K_2_ Q^2^_e_) + 1/Q_e_ t (5)

where Q_e_ is the adsorption capacity of HP on polyphenols at the equilibrium time (μg/mg HP), Q_t_ is the adsorption amount of HP on polyphenols at a certain time (μg/mg HP), K_1_ is the adsorption rate constant of pseudo-first order kinetic (min^−1^), K_2_ is the adsorption rate constant of pseudo-second order kinetic (min^−1^), t is the adsorption time (min).

#### 2.5.2. Adsorption Isotherm Model Fitting

The Langmuir model is a single-layer adsorption model and it is assumed that the solute molecules are saturated when the adsorption on the adsorbent surface reaches the maximum. It considers the influence of temperature on the experiment and can predict the maximum adsorption capacity (Q_m_). The Langmuir adsorption expression is:

Q_e_ = Q_m_ K_L_ C_e_/(1 + K_L_ C_e_) (6)

The Freundlich model is an empirical adsorption model to describe the different adsorption sites and adsorption energies on adsorbent surfaces. It can judge the adsorption performance of adsorbent. The Freundlich adsorption expression is:

Q_e_ = K_f_ C_e_^1/n^ (7)

where C_e_ is the polyphenol concentration at adsorption equilibrium (μg/mL), Q_e_ is the adsorption capacity of HP for polyphenol adsorption equilibrium (μg/mg HP), Q_m_ is the maximum adsorption capacity of HP, which is related to adsorption site (μg/mg HP), n is the Freundlich exponent, K_L_ and K_f_ are the adsorption constant [8].

### 2.6. Characterization of Interaction between HP and Polyphenols

#### 2.6.1. Turbidity Analysis

The turbidity of mixtures was measured at 650 nm on a 96-well microplate by using a microplate reader (SYNERGY-2, BioTek Instruments, Inc., Winooski, VT, USA). A series of EC and CA (0–0.35 mg/mL) and HP (0–3.5 mg/mL) were prepared in 0.1 M citrate/phosphate buffer (pH 3.8), respectively. Equal volumes of EC and CA were mixed HP, respectively and then the mixed system was constantly shaken at 100 r/min on an orbital shaker at room temperature. EC, CA and HP were dissolved in 0.1 M citrate/phosphate buffer (pH 3.8) respectively as control. All measurements were done in triplicate [12].

#### 2.6.2. Dynamic Light Scattering (DLS) Analysis

The average particle size, zeta potential and polydispersity index (PDI) of mixtures were measured by DLS at a Malvern Laser Particle Size Analyzer ZS90 (Malvern Instruments, Malvern, UK). To avoid the generation of bubbles, the sample was dispersed by ultrasonic for 5 min and slowly added into the sample cell. Afterwards, each sample was preheated to 25 °C for 30 s in water and tested three times in parallel [15].

#### 2.6.3. Scanning Electron Microscope (SEM) Analysis

The samples (HP, EC, CA, HP-EC mixture and HP-CA mixture) were deposited on the double-sided conductive adhesive for sample preparation and then gold spraying was carried out for 120 s with a current of 15 mA. After, images were observed using the upper electron microscope (Hitachi S-4800, Tokyo, Japan) at a working voltage of 5 KV and a current of 10 μA [32].

#### 2.6.4. UV-VIS Spectrum Analysis

The samples (HP, EC, CA, HP-EC mixture and HP-CA mixture) were recorded by a UV-VIS spectrophotometer (UV-1800, Shimadzu, Japan) from 190 nm to 500 nm [33].

#### 2.6.5. Fourier Transform Infrared Spectroscopy (FT-IR) Analysis

FT-IR spectra of samples were determined by a spectrometer at the spectral range of 4000 to 400 cm^−1^ for 32 scans with 4 cm^−1^ resolution (IRAffinity-1S, Shimadzu, Japan). Briefly, the 20 μL of sample solution (HP, EC, CA, HP-EC mixture and HP-CA mixture) was added to the dried KBr (200 mg), respectively, and then ground and pressed into a 1 mm pellet for FT-IR spectral analysis. The absorption spectra were obtained after denoising and baseline correction [34].

#### 2.6.6. Isothermal Titration Calorimetry (ITC) Analysis

The change of entropy, enthalpy and other binding parameters caused by the interaction between HP and polyphenols (EC and CA) were measured by ITC, using VP-ITC microcalorimeter (Malvern Instruments, Malvern, UK). HP (10 μM) and polyphenols (200 μM) were prepared in 0.1 M citrate/phosphate buffer (pH 3.8). The reference cell was filled by buffer while the titration cell of the calorimeter was loaded with 1.4 mL of HP solution. The EC and CA solution were loaded into injection syringe and titrated into the sample cell by 28 injections of 10 μL, respectively. Each injection lasted 180 s with an interval delay of 20 min. The content of the titration cell was constantly stirred throughout the experiments at 372 r/min. Raw data obtained as a plot of heat flow (microjoules per second) against time (minutes) were then integrated peak-by peak and normalized to obtain a plot of observed enthalpy change per mole of injectant (ΔH, KJ/mol) against the molar ratio. Peak integration was performed and the experimental data were fitted to a theoretical titration curve using NanoAnalyze v3.7.5 (TA Instruments, New Castle, DE, USA). Control experiments included the titration of EC and CA fractions into buffer respectively and were subtracted from titration experiments. The thermodynamic parameters including the number of binding sites per molecule (n), binding constant (Ka), enthalpy (ΔH) and entropy (ΔS). The thermodynamic parameters ΔG and ΔS were calculated from the van’t Hoff equation:

ΔG = −RT ln Ka = ΔH − TΔS(8)

where ΔG is free enthalpy, Ka is the association constant, ΔH is the enthalpy, and ΔS is the entropy of interaction [12,15].

### 2.7. Statistical Analysis

Microsoft Office Excel 2010 (Microsoft Corporattion, Washington, DC, USA) was used for statistical evaluation. Origin 2018 (Origin Lab, Northampton, MA, USA) was applied for fitting experimental data. Data are expressed as the mean ± standard deviation (S.D.). A one-way analysis of variance (ANOVA) was applied to analyze the variances for the adsorption studies of individual polyphenols. Duncan’s multiple comparison test was used to determine significant differences, at *p* < 0.05 using SPSS software version 19.0 (SPSS Inc., Chicago, IL, USA) [35].

## 3. Results

### 3.1. Characterization of Hawthorn Pectin

The results of preliminary characterization of HP showed that the uronic acid and total sugar content of HP was 57.81 ± 1.01% and 63.40 ± 0.28%, respectively. The degree of methyl esterification of HP was 54.95 ± 1.90% (Table S1). Thus, it could be primarily drawn that HP obtained by hot water extraction was high methoxyl pectin, which was consistent with our previous studies [3]. Whereas it was inconsistent with the ultrasonic-assisted enzymatic extraction of polysaccharide from flowers and fruits of hawthorn (Crataegus monogyna Jacq.) [36]. The reason for this difference was probably coming from the different varieties of hawthorn and extraction methods. The molecular weight (Mw) of HP was (2120 ± 59) KDa (Table S1). PMP derivatization and HPLC were used to detect the monosaccharide composition in HP (Table S2). The results showed that HP was mainly composed of galacturonic acid and glucose, followed by mannose, arabinose and rhamnose.

### 3.2. Adsorption Experiments Analysis of Different Polyphenols

#### 3.2.1. Adsorption Kinetics Analysis

##### Study on Adsorption Kinetics

**Figure 1:**
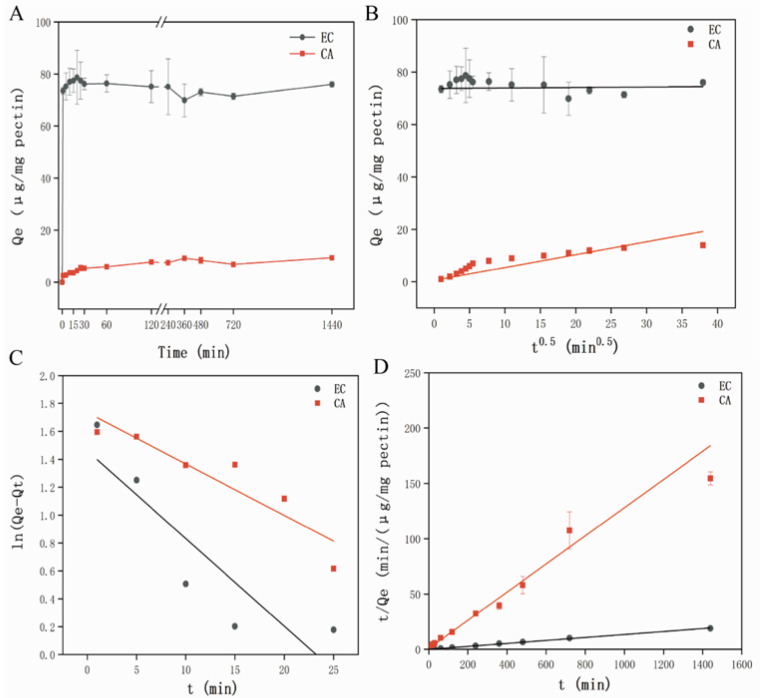
(**A**) Adsorption kinetics curves of EC and CA; (**B**) Intragranular diffusion equation fitting; (**C**) Pseudo-first order dynamic equation fitting; (**D**) Pseudo-second order dynamic equation. EC: epicatechin; CA: chlorogenic acid.

The binding of HP to two representative hawthorn polyphenols (EC and CA) were investigated. Figure 1A presented the binding of HP to EC and CA from 0 to 24 h. For EC, the adsorption proceeded rapidly after 1 min, then the adsorption capacity increased slowly with an increase in the contact time, and then a plateau was obtained from 2 to 4 h. After 4 h, no significant increase (*p* > 0.05) in binding was observed, agreed with previous studies [18,37]. For CA, the adsorption capacity increased rapidly after 1 min and then extended to 4 h and remained stable, which were consistent with previous studies [38]. After 4 h, the adsorption capacity slightly changed with the extension of time, although no significant difference (*p* > 0.05). Based on the time results, 4 h of contact time was selected for the later adsorption isotherm study.

Since the interactions between HP and polyphenols occurred spontaneously after 1 min and rapidly increased until 25 min, it could be inferred that plant-based food systems that were rich in polysaccharides and polyphenols such as fruits, vegetables and cereals were likely inevitable result in the combination of polysaccharides and polyphenols when they were subjected to physical damage, chewing and other external forces. These results were consistent with previous studies which reported the combination of apple cell wall and procyanidins based on different processing patterns within 1 min [7] and the binding between plant cell wall analogues and anthocyanins from purple carrot before 30 s of exposure [18].

Furthermore, it was observed that the adsorption capacity of HP to EC was about 9 times higher than that of CA (39.4% and 4.0% for EC and CA, respectively). We could infer that the equilibrium adsorption amounts for two polyphenols on HP followed the order of EC > CA. Liu et al. have suggested that the adsorption capacity of bacterial cellulose to EC at the plateau stage could reach 13.0%, which was much lower than our result [7]. Liu et al. have showed the maximum adsorption capacity of apple cell wall to EC and CA was 22% and 6.5%, respectively. The difference between previous studies and our findings, which may be related to the raw materials and adsorption conditions [8].

##### Adsorption Kinetics Model Fitting Analysis

The models of intragranular diffusion, pseudo-first order and pseudo-second order kinetic were the most known and applicable kinetic models of adsorption on the adsorbents [38]. These models could well reveal the adsorption process of pectin and polyphenols in this experiment and the model fittings and parameters calculation were shown in Figure 1 and Table S3, respectively. Figure 1B showed the straight line plots of Q_t_ vs. t^0.5^ and described the rate constant of intragranular diffusion (K_p_) and the correlation coefficient (R^2^). The straight line did not cross the origin which indicated that the fitting effect between HP and polyphenols (EC and CA) was not good, and other adsorption mechanisms may be accompanied by the interaction between pectin and polyphenols. As shown in Table S3, the correlation coefficients (R^2^) of EC and CA were 0.016 and 0.705, the rate constant of intragranular diffusion (K_p_) were 0.018 and 0.178, respectively. These investigations indicated that the fitting effect was not good.

Therefore, the pseudo-first and pseudo-second order adsorption models were adopted to explore other adsorption mechanisms and these fitting results were shown in Figure 1C,D, respectively. In addition, a presence of some negative values located at longer periods of the process were not showed in Figure 1C, which indicated that the pseudo-first order kinetic model did not fit the whole range of contact time was only applicable for the initial stage of the adsorption process [39]. The rate constants obtained from the plot of experimental data and the correlation coefficients of the two kinetic models were also shown in Table S3.

As shown in Table S3, the correlation coefficients (R^2^) of the pseudo-first order model for EC and CA were 0.793 and 0.853, which were relatively lower than the correlation coefficients (R^2^) of the pseudo-second order model (0.998 and 0.975 for EC and CA, respectively). It was obviously that the pseudo-second order kinetic model was a better fit than the pseudo-first order kinetic model. Moreover, the calculated Q_e_ value (75.188 and 8.873 μg/mg HP for EC and CA, respectively) was close to the experimental data (76.221 and 7.434 μg/mg HP for EC and CA, respectively) in the case of pseudo-second order kinetic model. These results indicated that the adsorptive process between HP and polyphenols followed a pseudo-second order kinetic, which was mainly controlled by chemical adsorption, agreed with the adsorption mechanism between chitosan and tannic acid [38].

#### 3.2.2. Adsorption Isotherm Models Analysis

Adsorption isotherm models were an important study method to determine the efficiency of the adsorption process. Langmuir and Freundlich isotherm models have been applied for fitting the experimental data. The model fittings and parameterrs calculation were shown in Figure S1 and Table S4. As shown in Figure S1, the amount of adsorption HP to polyphenols increased non-linearly with the increasing concentration of free polyphenols, whereas adsorption behavior varies may depend on different polyphenol classes. As a result, the number of aromatic moieties and phenolic hydroxyl groups presented in the chemical structure and neutral charge which might explain why HP bounds to EC with a greater extent than CA. Structurally, the representative of flavan-3-ols (EC) had two aromatic rings and four phenolic hydroxyl groups, whereas CA as the representative of phenolic acid had one aromatic ring and two phenolic hydroxyl groups. Previous studies have shown that the number of aromatic rings and hydroxyl groups (e.g., phenolic hydroxyl groups) presented in the molecular structures would contribute to the non-covalent interaction between polyphenols and cellulose, mainly through hydrophobic and hydrogen bonding [40,41]. Furthermore, more ortho phenolic hydroxyl groups and aromatic ring made for a greater number of combinations between polysaccharides and polyphenols [14]. In this study, lower adsorption between HP and CA also might be partly due to electrostatic interaction between HP and CA with negative charge.

Langmuir and Freundlich isotherm models were applied to predict the maximum apparent binding capacity (Q_m_) and the apparent binding affinity constant (K_L_) of different polyphenols, which are shown in Table S4. Both models could provide a good fit to experimental adsorption data (R^2^ > 0.97). The correlation coefficients (R^2^) of Langmuir and Freundlich isotherm adsorption model were 0.999 and 0.998, respectively, which could be seen that Langmuir isotherm adsorption model was slightly better than Freundlich isotherm adsorption model, agreed with Liu et al. [8]. The results indicated that the adsorption mechanism between HP and polyphenols was closed to the monolayer adsorption process, which was agreed with adsorption isotherm studies on the interaction between apple cell walls and polyphenols [8].

The maximum apparent binding capacity (Q_m_) could be interpreted as total number of binding sites that were available for adsorption. The Q_m_ of EC was 12 times higher than CA (293.627 ± 6.265 and 23.71 ± 3.440 μg/mg HP, respectively). The K_L_ of EC and CA was calculated (0.014 ± 0.0006 and 0.009 ± 0.0002, respectively) and did not show the same pattern, indicating that HP had different binding capacities to different polyphenols. Liu et al. have indicated that Q_m_ and K_L_ were used to represent for the binding saturation level and the binding affinity, respectively [8]. In fact, high binding capacities were always consistent with low binding affinities. Interestingly, the Q_m_ of EC was much higher than CA, whereas the affinity of EC and CA was similar. Furthermore, the fitting result is much higher than the actual adsorption capacity, agreed with previous studies [17,41].

#### 3.2.3. Effect of Solution Conditions on Adsorption Capacity of Different Polyphenols

**Figure 2:**
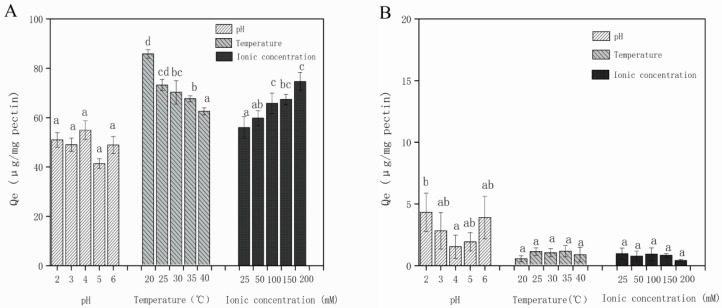
Effect of solution conditions on adsorption capacity of EC (**A**) and CA (**B**). Different letters represent significant differences (*p* < 0.05). EC: epicatechin; CA: chlorogenic acid.

For pH, it had different effects on EC and CA (Figure 2). With the change of pH, the adsorption amount of EC changed, although there was no significant difference (*p* > 0.05). Furthermore, the changing trend of CA with pH was different from that of EC. The adsorption amount decreased significantly only at pH 4 (*p* < 0.05), and then did not change significantly with the increase or decrease of pH (*p* > 0.05). Liu et al. recorded that the pKa of pectin and CA were 3.5 and 3.3 respectively [8]. When the pH was 4, both of them were negatively charged, therefore there was electrostatic repulsion. Theoretically, with the further increase of pH, the electrostatic interaction would be strengthened, and the adsorption amount would be further reduced correspondingly. However, our results had no significant change, so it could be inferred that there were other driving forces, and electrostatic interaction was not the main driving force.

For temperature, with the increase of temperature, the adsorption of HP and EC decreased slightly at 25 °C (*p* > 0.05) until it decreased significantly at 30 °C (*p* < 0.05), and then decreased continuously with the increase of temperature (Figure 2). However, the adsorption of HP and CA did not change significantly with the increase of temperature (*p* > 0.05). It could be seen that the interaction between HP and EC was more affected by temperature than CA. Phan et al. indicated that the interaction between polyphenols and polysaccharides would be weakened with the increase of temperature, which might be due to the fact that the temperature changed the stability of polyphenols in solution and the enthalpy of binding [41]. Increasing the temperature may improve the solubility of the polyphenol molecules in the aqueous solution, thereby limiting the combination of polyphenol with other substances. Previous studies showed that the temperature effect might be partly due to the hydrogen bond effect, and it might also be related to Van der Waals force, hydrophobic interaction, and π-π conjugate and other noncovalent interactions [14,42].

For ionic concentration, with the increase of ion concentration, the adsorption amount of HP and EC showed a significant difference until it reached 100 mM (*p* < 0.05), while there was no significant difference when the ionic concentration continually increased (*p* > 0.05). Nevertheless, the adsorption amount of HP and CA did not change significantly with the increase of ionic concentration (*p* > 0.05, Figure 2). Previous studies have shown that hydrophobic interaction has been established between hydrophobic regions of cellulose polymers and hydrophobic groups (such as aromatic rings) in the molecular structure of polyphenols. The interaction was enhanced with the increase of ionic strength (*p* > 0.05), which could be inferred that the influence of ionic concentration on adsorption amount was not dominant [14]. Our research results were consistent with previous researches and indicated hydrophobic interaction was not the main driving force.

Based on these results, it could be inferred that pH, temperature and ion concentration had different effects on different polyphenols. Our analysis showed that the interaction between HP and polyphenols might mainly involve hydrogen bond, hydrophobic interaction and electrostatic interaction, while hydrophobic and electrostatic interaction were not the main internal driving force, the main driving mechanism need to be further studied.

### 3.3. Turbidity Analysis

Figure S2 showed the variation in turbidity at 650 nm between pectin and polyphenols (EC and CA). The turbidity of the mixtures of HP and polyphenols rose with increasing HP concentrations, which may be due to the slight haze formation (Figure S2A). This increase was reaching a maximum at 3.2 mg/mL for EC and CA, with optical density (OD) values of 0.051 and 0.060, respectively. Figure S2B showed the optical density of HP with the different polyphenols concentrations. As the concentration of EC increased, the turbidity observed increased until reaching an equilibrium absorbance of 0.043 at 0.16 mg/mL. In contrast, with the increase of CA concentration, there was no significant difference in adsorption (*p* > 0.05). These behaviors showed that the interactions between pectin and polyphenols included the formation of insoluble aggregates, agreed with the subsequent the results of UV-VIS and FT-IR spectra. The results were consistent with previous studies [12,15].

### 3.4. DLS Analysis

**Figure 3:**
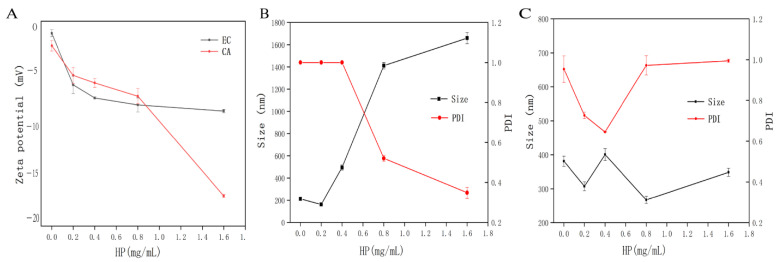
The average particle size and zeta potential between HP and polyphenols (ratio of 5:1). (**A**) The zeta potential between HP and polyphenols (EC and CA). (**B**)The average particle size and polydispersity index (PDI) between HP and EC. (**C**) The average particle size and polydispersity index (PDI) between HP and CA. HP: hawthorn pectin; EC: epicatechin; CA: chlorogenic acid.

ζ-potential is an important method to further understand the electrostatic interaction between polyphenols and polysaccharides. In addition, ζ-potential has a guiding significance for evaluating the long-term stability of complex system. Figure 3A showed that the potential values of EC and CA solutions alone were negative, and the number of negative charges of the mixture was more than that of the single solution itself. The initial potentials of EC and CA were −1.23 ± 0.36 and −2.51 ± 0.54 mV, respectively. With the increase of HP concentration, the potential of the mixture decreased (*p* < 0.05). When the concentration of HP increased to 0.4 mg/mL, the potential of HP-EC mixture reached the equilibrium potential of −7.86 ± 0.12 mV. However, the concentration of HP increased to 1.6 mg/mL, the HP-CA composite system reached the lowest potential of −17.10 ± 0.14 mV and it was not saturated. This may be because the potential of HP-CA composite system reached the saturation state with a higher potential, while the maximum addition of HP in this experiment still did not reach the saturation potential of the composite system. The saturation potential of HP-CA was higher than that of HP-EC. Furthermore, Mamet et al. have shown that the higher ζ-potential (positive or negative) represented a stronger intermolecular repulsion and the relatively stable system [15]. However, the HP-EC system with lower potential tends to condense or agglomerate, which meant the attraction force exceeded the repulsion force, which may be the reason that the adsorption capacity of HP for EC was much higher than that of CA, which was consistent with the results of adsorption kinetics. Vallar et al. showed that when the absolute value of particle potential was less than 30 mV, mixtures tended to aggregate, and showed weak stability [43]. On the contrary, when the absolute value of potential was greater than 30 mV, they would repel each other and formed a stable suspension with relatively uniform dispersion [43]. It could be seen that the potentials of HP-EC and HP-CA were all below 30 mV, which indicated that the complexes were unstable.

The particle size and PDI of EC and CA system before and after adding HP were observed by DLS. It could be seen from Figure 3B,C that the particle size of EC and CA was 212.35 ± 13.65 and 381.10 ± 15.41 nm, respectively. When 0.2 mg/mL HP was added to EC and CA system, respectively, the particle size decreased to 162.00 ± 4.38 (*p* > 0.05) and 307.47 ± 13.23 (*p* < 0.05) nm, which indicated that the addition of HP could better reduce the self-aggregation of CA compared with EC. In addition, with the increase of HP concentration, the particle size of HP-EC increased significantly (*p* < 0.05), which might be due to the formation of new aggregates between HP and EC, which led to the increase of particle size of the system. However, the result of the mixture of HP and CA was not obvious, which might be related to its own charge, resulting in the unstable combination. This result was consistent with the subsequent UV-VIS and turbidity analysis. Our results showed changes consistent with Mamet et al. [15], who studied the particle size of persimmon tannin and pectin.

Furthermore, the PDI of EC and CA solutions alone was 1 and 0.95, respectively. Low concentration of HP (0.2–0.4 mg/mL) significantly changed the PDI value of CA solution (*p* < 0.05), while high concentration of HP significantly reduced the PDI value of EC solution (*p* < 0.05). These results indicated that the addition of HP might interact with EC and CA in the system to make the solution system more uniform and obtain a composite solution with better dispersibility, which also explained that the addition of HP changed the states of EC and CA to some extent. With the addition of high concentration of CA, there might be a stronger electrostatic interaction in the system, and the system tended to be more stable [15]. Nevertheless, the PDI results showed that the PDI value of the system was higher at the higher the concentration of CA, which showed that electrostatic interaction was not the main force driving interaction. Our results were consistent with Carvalho et al. [5], who studied pectin-tannin-protein interactions.

### 3.5. SEM Analysis

**Figure 4:**
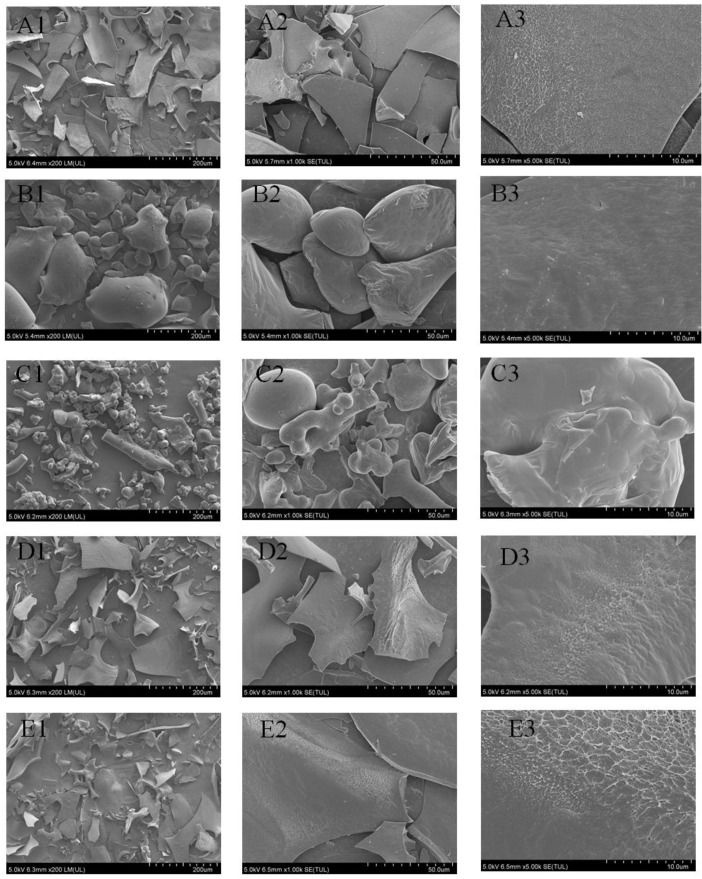
SEM pictures of HP (**A**), EC (**B**), CA (**C**), HP-EC (**D**), HP-CA (**E**); and the row exhibited different magnifications are at (1) 200×; (2) 1k×; (3) 5k×. HP: hawthorn pectin EC: epicatechin; CA: chlorogenic acid; HP-EC: the mixture of hawthorn pectin and epicatechin; HP-CA: the mixture of hawthorn pectin and chlorogenic acid.

The SEM is used to observe and record the surface morphological characteristics of HP, EC, CA, HP-EC and HP-CA (Figure 4). It was apparent that a difference existed in HP, EC, CA and HP-phenol mixtures, indicating that different types of polyphenols had characteristic ways of being incorporated within HP. The citrus peel pectin showed an irregular shape with a smooth surface [41], while apple pectin presented a honeycomb-like morphology filled with interconnected walls and large empty spaces [14]. According to the captured pictures (Figure 4), it was apparent that the microstructure of EC and CA was spherical and rod shaped, whereas HP showed a flaky structure with enough space, which could be occupied by different polyphenols [44]. The HP-phenol mixtures exhibited a more wrinkles surface rich in protrusions structure compared to HP (Figure 4D–E), which indicated that the addition of polyphenols had an effect on the surface morphology of HP. The change of surface morphology of HP might be due to the interaction between HP and polyphenols by inter- and intramolecular hydrogen bonds. The morphology features of pectin changed after binding with procyanidin/catechin [45]. The change in surface of arabinan-rich pectin was mostly attributed to binding with ferulic acid [33].

### 3.6. UV-VIS Spectrum Analysis

As shown in Figure S3, EC and HP-EC mixture both had signification absorption at around 275 nm, whereas the typical absorption peak of CA and mixture was at around 326 nm. Furthermore, all of these absorption peaks slightly decreased in the corresponding mixtures. Thus, these results implied that the interaction of HP and polyphenols, which changed the intensity of EC and CA peaks. Li et al. showed that catechin and gallic acid were significantly adsorbed by soluble dietary fiber, which were determined by UV-VIS and FT-IR spectra [44]. As reported in previous studies, polysaccharides had no absorption peak at 200–400 nm in common and the adsorption behavior between polyphenols and polysaccharides could be confirmed by comparing the UV spectra [46].

### 3.7. FT-IR Analysis

**Figure 5:**
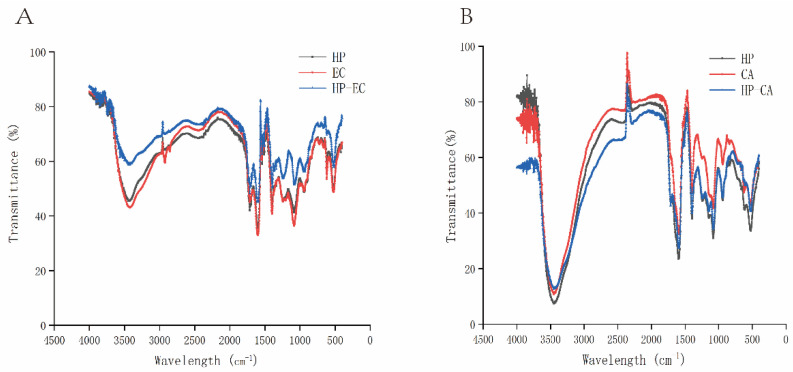
FT-IR spectrum of HP, EC and HP-EC mixture (**A**); CA and HP-CA mixture (**B**). HP: hawthorn pectin EC: epicatechin; CA: chlorogenic acid; HP-EC mixture: the mixture of hawthorn pectin and epicatechin; HP-CA mixture: the mixture of hawthorn pectin and chlorogenic acid.

The FT-IR spectra of HP, EC, CA and HP-polyphenols mixtures were showed in Figure 5. The characteristic peak of HP at 3000–3500 cm^−1^ was related to the hydrogen bonding of O-H group in galacturonic acid unit. The characteristic peak at 2924.09 cm^−1^ was related to the C-H bond of CH, CH_2_ and CH_3_ of galacturonic acid. The peaks at 1714.72 and 1606.70 cm^−1^ were corresponded to esterification and free carboxyl of galacturonic acid polymer. The peaks at 1000–1250 cm^−1^ were attributed to the C-O-C vibration in the glycosidic bond [44]. EC had strong absorption peaks at 3415.93, 1600.92 and 1074.35 cm^−1^, which were related to the characteristic functional groups of flavonoids [47]. For CA, the characteristic absorption peak of CA at 1589.34 cm^−1^. The absorption peak at 3446.79 cm^−1^ was the absorption peak of intermolecular hydrogen bond -OH group extensional vibration. The absorption peak at 589.34 cm^−1^ was caused by unsaturated -C=C- vibration. The absorption peak at 194.53 cm^−1^ was caused by C-H bending vibration. The absorption peak at 149.87 cm^−1^ was due to the vibration of C-O in carboxyl, which indicated the existence of carboxyl in molecule. The absorption peak at 1083.99 cm^−1^ was caused by C-O vibration, which indicated that the molecule contains phenolic hydroxyl and alcoholic hydroxyl [48]. Compared with the absorption peaks of EC and HP alone, it could be seen that the absorption intensity was obviously reduced from 3421.72 cm^−1^ to 3419.79 cm^−1^, which indicated that the O-H broadening was reduced due to the interaction between HP and EC. The results were consistent with Liang et al. [45], who confirmed that the molecular interaction between pectin and catechin/procyanidins could reduce the width of O-H bond. Furthermore, the absorption peak at 1074.35 cm^−1^ shifts to 1072.42 cm^−1^ was observed, which may be due to the formation of hydrogen bonds between the hydroxyl groups of polyphenols and pectin [45,49]. It was worth noting that the disappearance of the absorption peak at 621.07 cm^−1^ also suggested that there was an interaction between HP and EC, which may be a chemical combination. Compared with the peaks of HP and EC, there was no new peak in the interaction peak, which indicated that the interaction did not produce covalent bonds, which may be driven by hydrophobic interaction and hydrogen bonds [49]. CA was consistent with the change of absorption peak after the interaction of EC and HP. Combined with the decrease of interaction absorption peak in UV-VIS spectrum and the disappearance of the absorption peak, all of these could be speculated that chemical combination had taken place.

### 3.8. ITC Analysis

Both turbidity and dynamic light scattering measurements showed that HP could interact directly with EC/CA and form precipitate. Therefore, the ITC microcalorimeter was used to investigate the enthalpy changes in contact with interactions between HP and polyphenols at room temperature. The thermodynamic parameters from ITC titration of HP by polyphenols were shown in Table 1. The stoichiometry values of HP-EC and HP-CA were 6.32 (1 molecule of pectin bound with 0.16 molecule EC) and 15.4 (1 molecule of pectin bound with 0.06 molecule CA), respectively, using a one-site model. Additionally, the association constant obtained for EC was slightly higher than that of CA, which revealed a higher affinity between HP and EC than CA. Analysis of the thermodynamic contributions suggested an enthalpy contribution related to the exothermic interaction. Moreover, the entropy contribution for polyphenols was negative indicating that the interactions between HP and polyphenols were mostly driven by enthalpy, which mostly might involve hydrogen bonds and Van der Waals forces [12,15]. Furthermore, the values of both ΔH and ΔS were negative, indicating that the hydrogen bonds were involved in the interactions between HP and polyphenols. The free enthalpy (ΔG) was negative, which demonstrated spontaneous interactions between HP and polyphenols. The ITC results supported the previously discussed speculation of the primary driving forces and demonstrated that the interactions were primarily driven by hydrogen bonds and van der Waals forces, while hydrophobic and ionic interactions were not dominant.

## 4. Conclusions

The present study discussed the interactions between HP and polyphenols (EC and CA) in hawthorn juice simulation system. The binding adsorption behavior between HP and polyphenols was better fitted by pseudo-second order dynamic and Langmuir models, which indicated that the adsorption behavior was a monolayer adsorption behavior driven by chemical action. Meanwhile, pH, temperature and ion concentration also affected the intensity of interaction between HP and polyphenols. Stronger association was observed between HP and EC compared to CA, which may depend on the charge and structural characteristics. HP-EC and HP-CA exhibited higher turbidity characteristics, particle size and lower ζ-potential than EC and CA alone. The interaction between HP and polyphenols were mainly through hydrogen bonds and van der Waals forces, whereas electrostatic and hydrophobic interactions were not dominant. Based on this, the future work would focus on how to use the interaction between hawthorn pectin and polyphenol to improve the turbidity stability and unpleasant sensory experiences of real hawthorn juice production process, additionally, the interaction on the bioavailability of polyphenol would be also identified in the future.

## Figures and Tables

**Table 1:** Binding and thermodynamic parameters for the interaction between the different polyphenols with HP.

Parameters	HP-EC^1^	HP-CA^2^
n	6.32	15.40
K_a_ (M^−1^)	2.54 × 10^6^	2.09 × 10^6^
ΔH (cal·mol^−1^)	−1.11 × 10^6^	−1.12 × 10^6^
ΔS (cal·mol^−1^·K^−1^)	−336	−344
−TΔS (cal·mol^−1^)	1.02 × 10^5^	1.04 × 10^5^

HP-EC^1^ represents the mixture of hawthorn pectin and epicatechin. HP-CA^2^ represents the mixture of hawthorn pectin and chlorogenic acid.

## Data Availability

Data is contained within the article and Supplementary Materials.

## Supplementary Materials

The following supporting information can be downloaded at: https://www.mdpi.com/article/10.3390/foods11182813/s1, Table S1: Preliminary characterization of HP; Table S2: Analysis of HPLC results of HP; Table S3: Parameters of different adsorption kinetic models; Table S4: Parameters of different adsorption isotherm models; Figure S1: Adsorpption curves of EC and CA on HP fitting by (A) Langmuir isotherm model and (B) Freundlich isotherm model; Figure S2: The turbidity characteristics of interaction between HP and polyphenols (EC and CA). The measure of absorbance at 650 nm after interactions in 0.1 M citrate/phosphate buffer pH 3.8 (in triplicate). (A) Variation of absorbance of HP at different concentrations with polyphenols (0.08 mg/mL). (B) Variation of absorbance of polyphenols at different concentrations with HP (0.4 mg/mL); Figure S3: UV-VIS scanning results of HP, EC and HP-EC mixture (A); CA and HP-CA mixture (B).

## Abbreviations

HP	hawthorn pectin
EC	epicatechin
CA	chlorogenic acid
HP-EC mixture	the mixture of hawthorn pectin and epicatechin
HP-CA mixture	the mixture of hawthorn pectin and chlorogenic acid.
