# Supplementary material for: Hawthorn Juice Simulation System for Pectin and Polyphenol Adsorption Behavior: Kinetic Modeling Properties and Identification of the Interaction Mechanism
Source: Foods. 2022 Sep 13;11(18):2813. doi: 10.3390/foods11182813 (PMC9498233; doi:10.3390/foods11182813)
Supplement: Supplementary file 1 [file foods-11-02813-s001.zip › foods-1870210-supplementary.pdf]

Supplementary Material

Hawthorn Juice Simulation System for Pectin and Polyphenol Adsorption Behavior: Kinetic Modeling Properties and Identification of the Interaction Mechanism

Xuan Zhang [1], Meijiao Li [1], Wen Zhao [1,2], Zhe Gao [1], Mengying Wu [1], Tong Zhou [1], Chen Wu [1], Kaixuan Zhou [1], Xue Han [1] and Qian Zhou [1,2,*]

1 College of Food Science and Technology, Hebei Agricultural University, Baoding 071000, China

2 Engineering Technology Research Center for Agricultural Product Processing of Hebei, Baoding 071000, China

** Correspondence: zhouqian@hebau.edu.cn (Q.Z.); Tel.: +86-031-27528195 (Q.Z.)*

Table S1. Preliminary characterization of HP

Measurement index	Regression equation	R^2	Result
Total sugar (%)	y = 0.004x + 0.046	0.998	63.40 ± 2.75
Methyl esterification (%)	-	-	54.95 ± 1.90
Urionic acid (%)	y = 0.005x - 0.058	0.995	57.81 ± 1.01
Mw (KDa)	-	-	2120 ± 59

- indicates not detected.

Table S2. Analysis of HPLC results of HP

Sugar	Peak time (min)	Regression equation	Correlation coefficient (R^2)	Content (μg/mg HP)
Mannose	20.049	Y= 66.815 X -15.062	0.9990	4.80 ± 0.51a
Rhamnose	26.673	Y=58.182X + 92.079	0.9997	0.24 ± 0.05a
Galacturonic acid	33.685	Y= 64.367 X - 214.57	0.9925	29.32 ± 2.34b
Glucose	40.944	Y= 57.844X -180.87	0.9939	106.43 ± 4.44c
Galactose	46.36	Y= 69.468 X - 298.32	0.9954	-
Arabinose	50.249	Y=78.123X - 151.78	0.9978	10.50 ± 0.79a
Fucose	57.217	Y= 40.051 X +911.74	0.9899	-

- indicates not detected.

Different letters represent significant differences ($P < 0.05$).

Table S3. Parameters of different adsorption kinetic models

Polyphenols	Intragranular diffusion		Pseudo-first order		Pseudo-second order		
	K_p (μg·(mg·min$^{0.5}$)$^{-1}$)	R^2	K_1 (min^{-1})	R^2	Q_e (μg·mg^{-1})	K_2 (min^{-1})	R^2
EC[1]	0.018	0.016	0.063	0.793	75.188	3.258×10^{-3}	0.998
CA[2]	0.178	0.705	0.037	0.853	8.873	4.000×10^{-3}	0.975

[1]EC represents epicatechin.

[2]CA represents chlorogenic acid.

Table S4. Parameters of different adsorption isotherm models

Polyphenols	Langmuir isotherm model			Freundlich isotherm model		
	Q_m (μg/mg)	K_L	R^2	K_f	n	R^2
EC[1]	293.627 ± 6.265	0.014 ± 0.0006	0.999	7.872 ± 0.410	1.436 ± 0.030	0.998
CA[2]	23.710 ± 3.440	0.009 ± 0.0002	0.987	0.488 ± 0.097	1.454 ± 0.106	0.984

[1]EC represents epicatechin.

[2]CA represents chlorogenic acid.

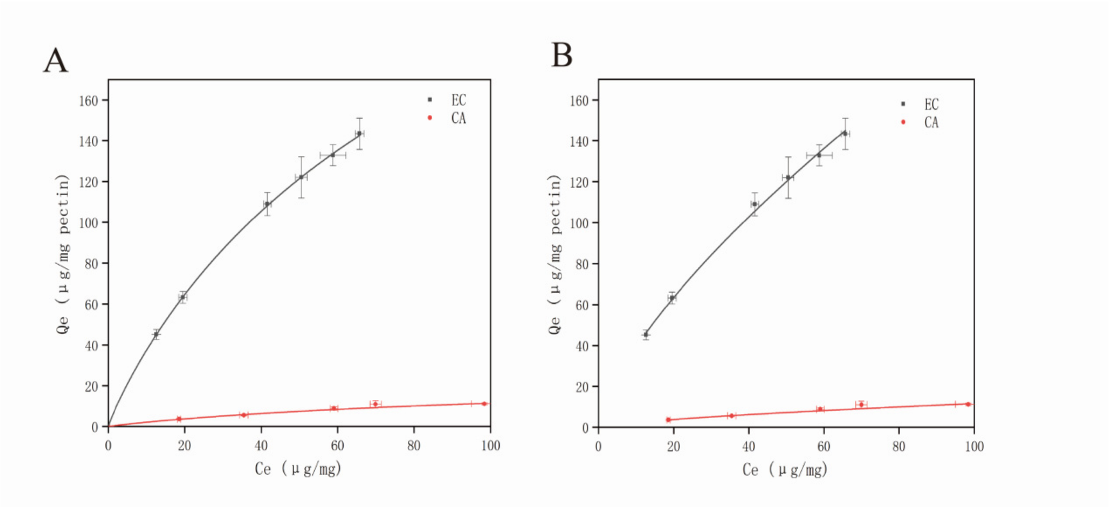

Figure S1. Adsorpption curves of EC and CA on HP fitting by (A) Langmuir isotherm model and (B) Freundlich isotherm model.

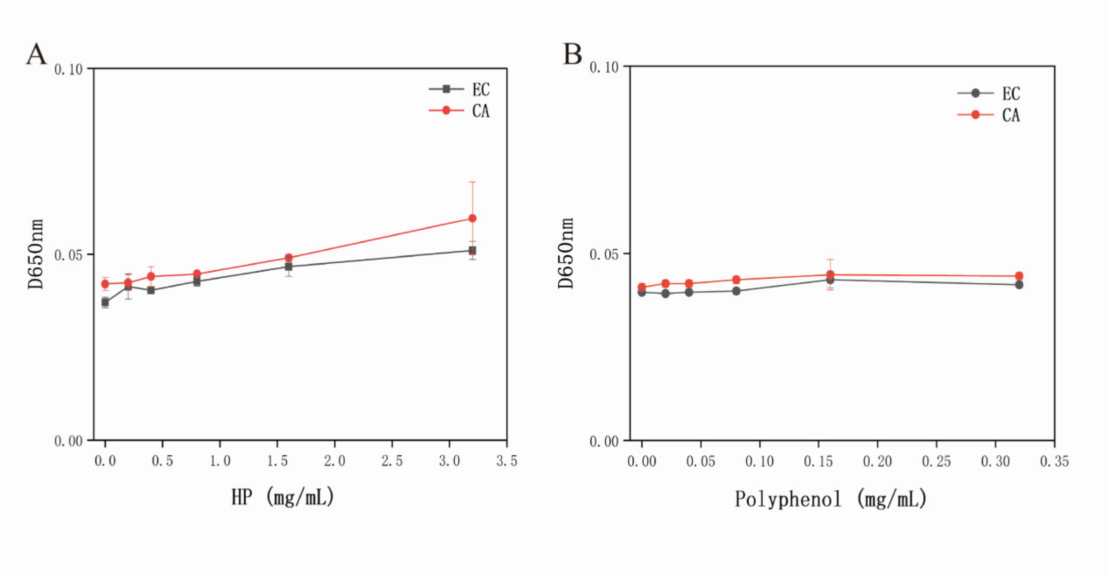

Figure S2. The turbidity characteristics of interaction between HP and polyphenols (EC and CA). The measure of absorbance at 650 nm after interactions in 0.1 M citrate/phosphate buffer pH 3.8 (in triplicate). (A) Variation of absorbance of HP at different concentrations with polyphenols (0.08 mg/mL). (B) Variation of absorbance of polyphenols at different concentrations with HP (0.4 mg/mL).

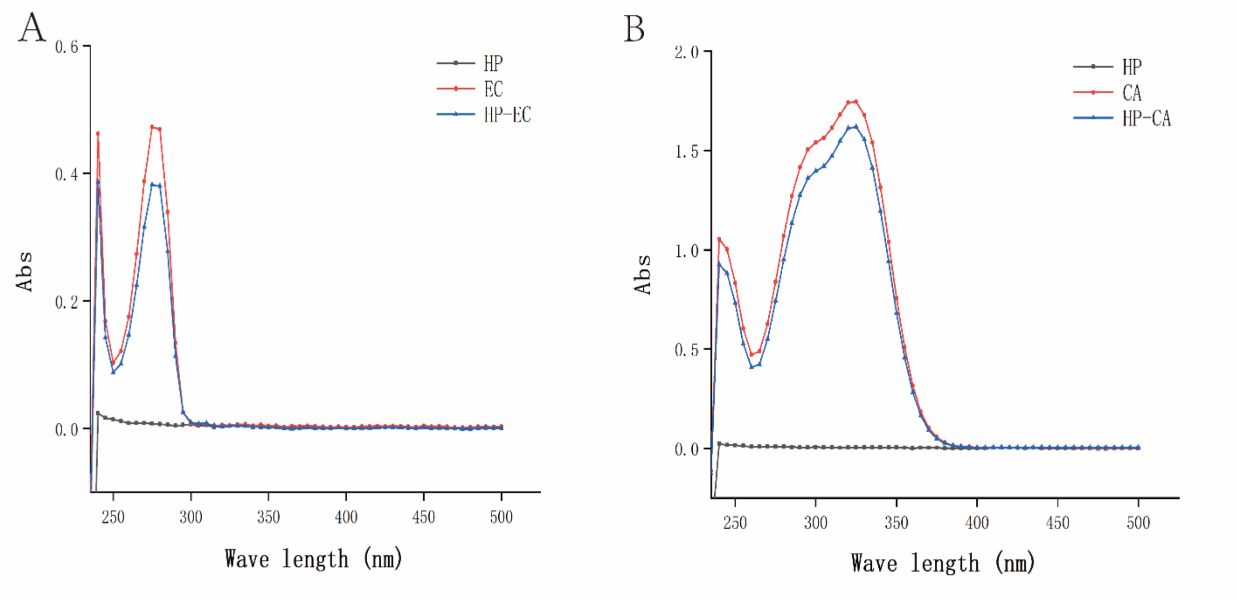

Figure S3. UV-VIS scanning results of HP, EC and HP-EC mixture (A); CA and HP-CA mixture (B).
